# Algorithmic Determinants of Performance Heterogeneity in Whole-Genome Sequencing-Based Prediction of Drug Resistance in *Mycobacterium tuberculosis*: A Systematic Review and Meta-Analysis

**DOI:** 10.3390/microorganisms14081824

**Published:** 2026-08-18

**Authors:** Baozhen Peng, Yang Zhou, Xiangchen Li, Huihui Liu, Bing Zhao, Ping Hou, Xichao Ou, Yanlin Zhao

**Affiliations:** 1 National Key Laboratory of Intelligent Tracking and Forecasting for Infectious Diseases, National Center for Tuberculosis Control and Prevention, Chinese Center for Disease Control and Prevention & Chinese Academy of Preventive Medicine, Beijing 102206, Chinazhouyang@chinacdc.cn (Y.Z.); zhaobing@chinacdc.cn (B.Z.); 2 Chinese Field Epidemiology Training Program, Chinese Center for Disease Control and Prevention & Chinese Academy of Preventive Medicine, Beijing 100050, China; liuhh@chinacdc.cn; 3 Cosmos Wisdom Mass Spectrometry Center, Zhejiang University Medical School, Hangzhou 310058, China; lxc@cwmda.com; 4 Tuberculosis Reference Laboratory, Hainan Provincial Center for Disease Control and Prevention, Haikou 570203, China; 18589565678@163.com

**Keywords:** *Mycobacterium tuberculosis*, whole-genome sequencing, drug resistance prediction, diagnostic accuracy, machine learning, bioinformatic tools, phenotypic drug susceptibility testing, meta-analysis

## Abstract

Whole-genome sequencing (WGS) is an increasingly adopted platform for predicting drug resistance in *Mycobacterium tuberculosis*; however, diagnostic accuracy varies substantially across bioinformatic tools and analytical frameworks, generating considerable uncertainty for clinical laboratory implementation. We conducted a prospectively registered (PROSPERO: CRD420261342739), PRISMA-DTA-compliant systematic review and meta-analysis of diagnostic accuracy studies. PubMed (MEDLINE), Embase, Web of Science, and Cochrane CENTRAL were searched from 1 January 2000 through 28 January 2026. Primary overall sensitivity and specificity were estimated using a tool-level bivariate random-effects model. Exploratory subgroup analyses and meta-regression examined the association between algorithm category and diagnostic-performance heterogeneity. Twenty-eight drug-level evaluations from seven tools (rifampicin, isoniazid, ethambutol, and pyrazinamide for each tool) were compiled from the extracted 2 × 2 data. For the primary tool-level composite analysis, pooled sensitivity was 0.930 (95% CI: 0.907–0.948) and pooled specificity was 0.962 (95% CI: 0.929–0.981). In secondary drug-specific analyses, sensitivity was highest for rifampicin (0.960, 95% CI: 0.934–0.976) and isoniazid (0.933, 95% CI: 0.906–0.953), and lowest for pyrazinamide (0.860, 95% CI: 0.800–0.904). Exploratory tool-level comparisons produced pooled sensitivity estimates of 0.920 for rule-based tools, 0.899 for machine learning tools, and 0.951 for hybrid tools. These comparisons involved only seven tool-level analytic units and cannot disentangle algorithm type from individual tool identity, training data, mutation catalogue version, or validation population. WGS-based bioinformatic tools provide highly specific and generally sensitive predictions of *Mycobacterium tuberculosis* resistance for first-line drugs across diverse clinical settings. Exploratory differences between tool categories should not be interpreted as causal effects of algorithmic architecture. Future studies should use prospective head-to-head evaluations on shared, geographically diverse isolate collections, alongside continued improvement of resistance catalogues and external validation.

## 1. Introduction

Tuberculosis (TB), caused by *Mycobacterium tuberculosis*, remains the leading infectious disease cause of mortality worldwide, with an estimated 1.23 million deaths among approximately 10.7 million incident cases in 2024 [1]. The disease burden is disproportionately concentrated in low- and middle-income countries (LMICs), where access to rapid and accurate diagnostic services remains severely constrained.

Drug-resistant tuberculosis (DR-TB)—encompassing rifampicin-resistant TB (RR-TB) and multidrug-resistant TB (MDR-TB)—substantially amplifies this public health crisis. Delayed or inaccurate resistance detection prolongs patient exposure to ineffective therapy, facilitates onward transmission, and increases case-fatality rates [2,3]. Phenotypic drug susceptibility testing (pDST), the current reference standard, requires weeks to months owing to the slow growth rate of *M. tuberculosis* and demonstrates variable inter-laboratory reproducibility, particularly for pyrazinamide and second-line agents [4,5]. Although targeted molecular assays—including the Xpert MTB/RIF platform, Xpert MTB/RIF Ultra, Xpert MTB/XDR, Truenat MTB/MTB-RIF, and line probe assays (LPAs) (including second-line LPA)—enable rapid detection of resistance to a limited drug set, with Xpert MTB/XDR and second-line LPA extending detection to fluoroquinolone, isoniazid, and injectable-agent resistance and thereby contributing, within their targeted scope, to pre-XDR/XDR-TB case-finding, none provide the comprehensive resistance profiling required to guide treatment of pre-extensively drug-resistant (pre-XDR) or XDR-TB [6,7].

Whole-genome sequencing (WGS) addresses these limitations by enabling simultaneous, unbiased detection of resistance-associated variants across the *Mycobacterium tuberculosis* genome from a single sequencing run [8]. The clinical utility of WGS has been substantially enhanced by the CRyPTIC (Comprehensive Resistance Prediction in Tuberculosis) genotype–phenotype dataset and the WHO catalogue of mutations in the *Mycobacterium tuberculosis* complex [4,9,10]. CRyPTIC provides paired sequence and minimum inhibitory concentration data that support discovery and validation of resistance-associated variants, whereas the WHO catalogue provides an expert-graded framework for interpreting variants associated with drug resistance [10]. Beyond resistance prediction, whole-genome sequencing can also distinguish relapse from reinfection in patients with recurrent tuberculosis, informing clinical management and epidemiological follow-up [11]. 

WGS-based resistance prediction is operationalised through bioinformatic tools that translate raw sequencing reads into clinically actionable resistance reports. These tools can be broadly classified into three analytical categories. Rule-based tools infer resistance by matching detected variants against predefined mutation catalogue or expert-curated decision rules, as exemplified by TB-Profiler and GenoMycAnalyzer. Machine learning tools derive predictive relationships from empirical genotype–phenotype data and include conventional statistical learning models as well as deep learning architectures, treated here as a subtype of the machine learning category throughout the quantitative synthesis. Hybrid tools, including Mykrobe (current stable release v0.13.0; version used by the included study not separately recorded), SAM-TB (web-based platform (https://samtb.uni-medica.com); specific version used by each included study not separately recorded), and tbtAMR (version not reported by the source study), combine curated resistance knowledge with data-driven modelling or probabilistic inference. This three-category framework was applied consistently in the quantitative synthesis [12,13,14,15,16,17,18].

Despite rapid expansion in clinical use, reported diagnostic accuracy varies substantially across studies—with sensitivity ranging from approximately 0.52 to 1.00 depending on drug, tool, and analytical approach—creating considerable uncertainty for laboratory directors and TB programme managers seeking evidence-based tool selection guidance [12,19].

Previous systematic reviews have characterised aggregate diagnostic accuracy or benchmarked individual tools; however, the determinants of performance heterogeneity remain incompletely understood. Tagami et al. (2024) and Chen et al. (2025) each reported aggregate diagnostic accuracy across multiple tools but did not formally test biological versus computational sources of heterogeneity via pre-registered meta-regression [3,20]. Specifically, it remains unclear whether variability primarily reflects biological factors—such as the genetic architecture and mutational complexity of drug-specific resistance mechanisms—or computational factors.

Resolving this question has critical practical implications: if variability is primarily biological, expanding mutation knowledge bases and deepening mechanistic understanding are paramount; if primarily computational, methodological advances and enhanced training data representativeness may rapidly improve performance.

We therefore conducted a systematic review and meta-analysis of diagnostic accuracy studies to: (i) estimate the overall and drug-specific accuracy of WGS-based tools for predicting *Mycobacterium tuberculosis* resistance to first-line drugs and (ii) explore descriptive patterns of sensitivity heterogeneity across the tools studied. Comparisons by algorithm category were prespecified as exploratory and are interpreted as hypothesis-generating because algorithm category is inseparable from the identity and characteristics of the individual tools in this evidence base.

## 2. Materials and Methods

### 2.1. Study Design and Registration

This systematic review and meta-analysis of diagnostic accuracy studies was conducted in accordance with the Preferred Reporting Items for Systematic Reviews of Diagnostic Test Accuracy (PRISMA-DTA) guidelines [21] and was prospectively registered in PROSPERO prior to data extraction (registration number: CRD420261342739). The primary objective was to quantify the diagnostic accuracy of WGS-based bioinformatic tools for predicting *M. tuberculosis* drug resistance, using pDST as the reference standard. Secondary objectives were investigation of heterogeneity sources through prespecified subgroup analyses and meta-regression, with emphasis on analytical approach, drug class, and study size.

Throughout this report, ‘study’ denotes a unique included publication, and ‘dataset’ denotes unique extractable results within a study, defined by drug or resistance outcome and, where applicable, the specific analytical model employed.

### 2.2. Literature Search Strategy

We searched PubMed (MEDLINE), Embase, Web of Science Core Collection, and the Cochrane Central Register of Controlled Trials (CENTRAL) from 1 January 2000 through 28 January 2026, employing controlled vocabulary terms (MeSH for PubMed; Emtree for Embase) in combination with free-text keywords related to *Mycobacterium tuberculosis*, whole-genome sequencing, drug resistance, and diagnostic accuracy. No language restrictions were applied. Reference lists of included studies and relevant systematic reviews were manually screened; clinical trial registries (WHO ICTRP and ClinicalTrials.gov) were searched to identify unpublished or ongoing studies. Full search strategies for all databases, including the exact Boolean query strings and database-specific syntax, are provided in Supplementary File S1.

An AI-assisted prescreening step (DeepSeek, DeepSeek-AI, Hangzhou, China) was used to expedite identification of clearly irrelevant records after the initial database searches. All AI-flagged exclusions were re-examined independently by two human reviewers against the full eligibility criteria, and no final inclusion or exclusion decision was made solely by AI. The original prompt, exact model version/date, and record-level audit trail of individual decisions and any reinstatements were not retained.

### 2.3. Study Selection Criteria

Two reviewers independently screened titles and abstracts, followed by full-text review, against predefined eligibility criteria; discrepancies were resolved by discussion or third-reviewer arbitration. Studies were eligible if they: (i) evaluated clinical isolates or patient-derived *M. tuberculosis* samples; (ii) employed WGS-based approaches with any bioinformatic analytical framework to predict drug resistance; and (iii) used pDST or culture-based methods as the reference standard. For inclusion in the quantitative meta-analysis, studies were additionally required to report data enabling reconstruction of a 2 × 2 contingency table (true positives, false positives, false negatives, true negatives). Studies exclusively evaluating laboratory-engineered mutants, non-clinical isolate collections, or computationally simulated genomes were excluded.

### 2.4. Data Extraction and Verification

Data extraction was performed independently by two reviewers using standardised, pre-piloted forms capturing study characteristics (publication year, geographic region, sample size, and *Mycobacterium tuberculosis* lineage distribution), methodological features (analytical approach, tool or pipeline, mutation catalogue source and version, pDST method, and laboratory accreditation), and 2 × 2 contingency data. For studies reporting results for multiple drugs, resistance categories, or analytical approaches, data were extracted at the dataset level, defined as unique study–drug–algorithm combinations. A second reviewer verified all extractions for the seven studies contributing quantitative data. Study-level descriptive characteristics for the remaining narrative-synthesis-only studies were compiled from the available reports and are presented in Supplementary Table S3; these data were not used in the quantitative meta-analysis. Discrepancies in the quantitative extractions were resolved by consensus or, where necessary, third-reviewer adjudication. Supplementary Table S1 reports the source citation and data-provenance category for every record, and Supplementary File S3 provides the statistical re-analysis. Full extracted variable details are provided in Supplementary Tables S2 and S3.

### 2.5. Risk-of-Bias and Methodological Quality Assessment

Risk-of-bias and applicability concerns were assessed independently by two reviewers using the Quality Assessment of Diagnostic Accuracy Studies-2 (QUADAS-2) tool across four domains: patient selection, index test, reference standard, and flow and timing. Overall risk-of-bias and applicability concerns were graded as low, unclear, or high for each domain. Disagreements were resolved by discussion and consensus. Domain-level and overall QUADAS-2 assessments for all 19 included studies, including reviewer-level assessments and consensus judgements, are provided in the updated QUADAS-2 workbook.

### 2.6. Statistical Analysis

For the primary analysis, pooled sensitivity and specificity were estimated using a bivariate random-effects logistic regression model fitted to seven tool-level composite 2 × 2 tables. This model jointly estimated sensitivity and specificity and their between-tool correlation.

The quantitative dataset comprised 28 drug-level diagnostic accuracy evaluations, including rifampicin, isoniazid, ethambutol, and pyrazinamide for each of seven tools. For the primary overall analysis, the four drug-specific 2 × 2 tables were summed within each tool to form one composite table per tool. The primary bivariate model was therefore fitted to seven independent tool-level units; the 28 drug-level tables were not treated as 28 independent studies. Drug-specific estimates were obtained in secondary univariate random-effects analyses across seven tools per drug. The comparison by algorithm category was conducted at the tool level and interpreted as descriptive and exploratory. Deeks’ funnel plot asymmetry testing was conducted only as an exploratory assessment of the 28 clustered drug-level records, with a 0.5 continuity correction applied to the single dataset containing a zero cell. Leave-one-tool-out analyses excluded one composite tool-level table at a time. All analyses were performed in R version 4.5.2 using metafor version 4.8-0; the analytical dataset and R scripts used to generate Figures 2–4 are provided in Supplementary File S3.

## 3. Results

### 3.1. Study Selection and Characteristics

**Figure 1:**
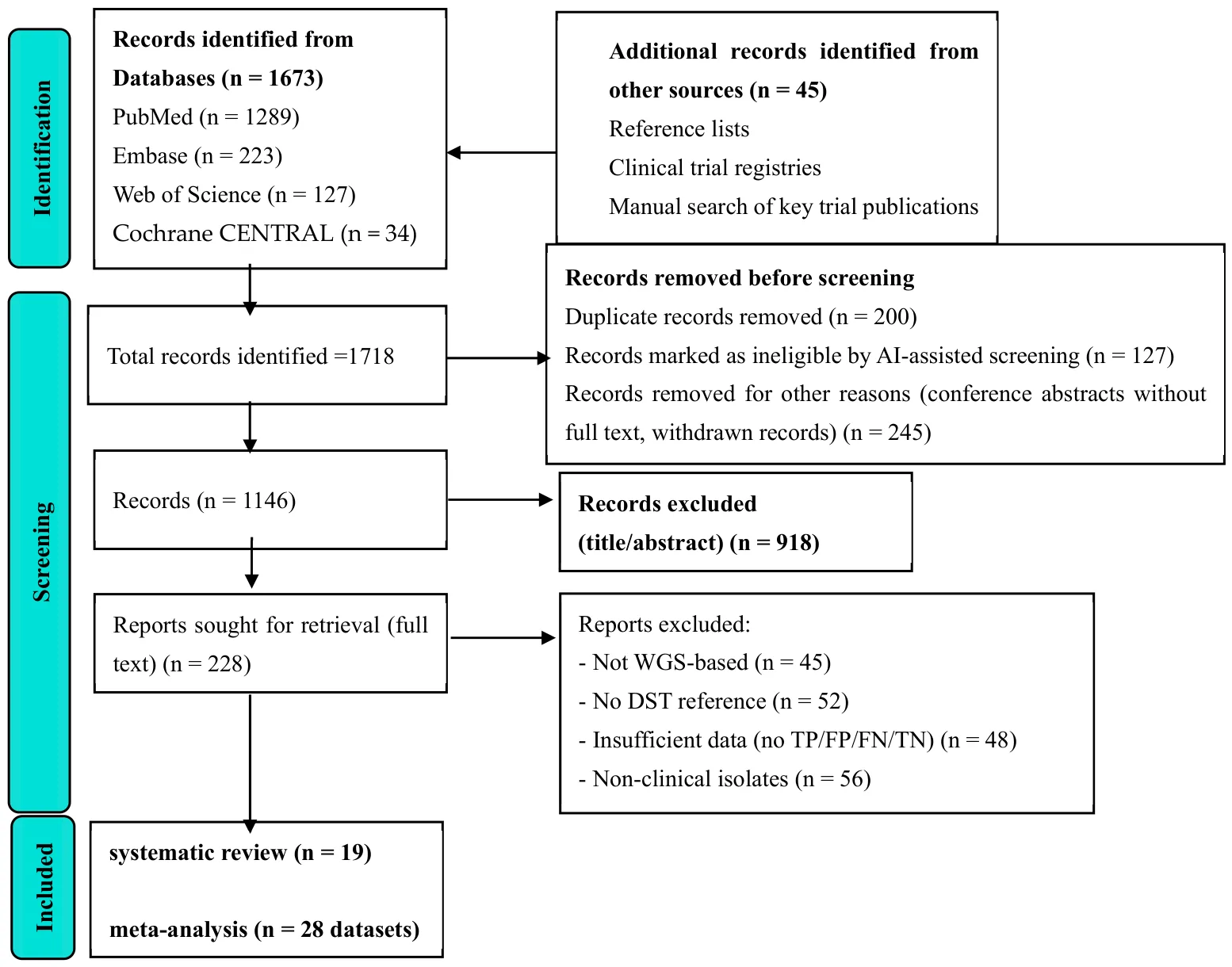
PRISMA 2020 flow diagram; terminal box updated to read “meta-analysis (*n* = 28 datasets)” in place of “meta-analysis (*n* = 13 datasets)”, consistent with the quantitative synthesis.

Systematic database searches identified 1673 records; manual searching of reference lists and clinical trial registries contributed 45 additional citations, yielding 1718 total records. After removal of duplicates (*n* = 200), AI-assisted title-level prescreening exclusions independently verified by two human reviewers (*n* = 127), and removal of conference abstracts without full-text and withdrawn records (*n* = 245), 1146 records underwent dual independent title-and-abstract screening. Of these, 918 were excluded against predefined criteria. Of the 228 reports assessed by full text, 209 were excluded for the following reasons: non-WGS methodology (*n* = 45), absence of a phenotypic reference standard (*n* = 52), insufficient data for 2 × 2 contingency table reconstruction (*n* = 48), or exclusive use of non-clinical isolate collections (*n* = 56), or a citation/identity that could not be independently verified against a matching primary source at data extraction (n = 8). Ultimately, 19 studies met criteria for qualitative synthesis, of which seven contributed 28 extractable drug-level datasets to the quantitative meta-analysis (Figure 1).

The 19 included studies spanned Asia, Europe, Africa, and multi-country settings, with cohort sizes ranging from 37 to 36,385 *M. tuberculosis* isolates (Table 1). Substantial methodological heterogeneity was observed across analytical frameworks, mutation catalogues employed, pDST methods, drug panels assessed, and data reporting granularity. Full study-level methodological characteristics are provided in Supplementary Table S3.

The seven tools/studies contributing quantitative data were Hunt et al. (2019) [22], Phelan et al. (2019) [12], Gröschel et al. (2021) [14], Yang et al. (2022) [15], Kim et al. (2024) [16], Wang et al. (2024) [17], and Horan et al. (2025) [18], collectively providing 28 drug-level evaluations (rifampicin, isoniazid, ethambutol, and pyrazinamide for each tool) encompassing rule-based, machine learning, and hybrid approaches. The remaining 12 studies contributed to the narrative synthesis.

### 3.2. Overall Diagnostic Accuracy

**Figure 2:**
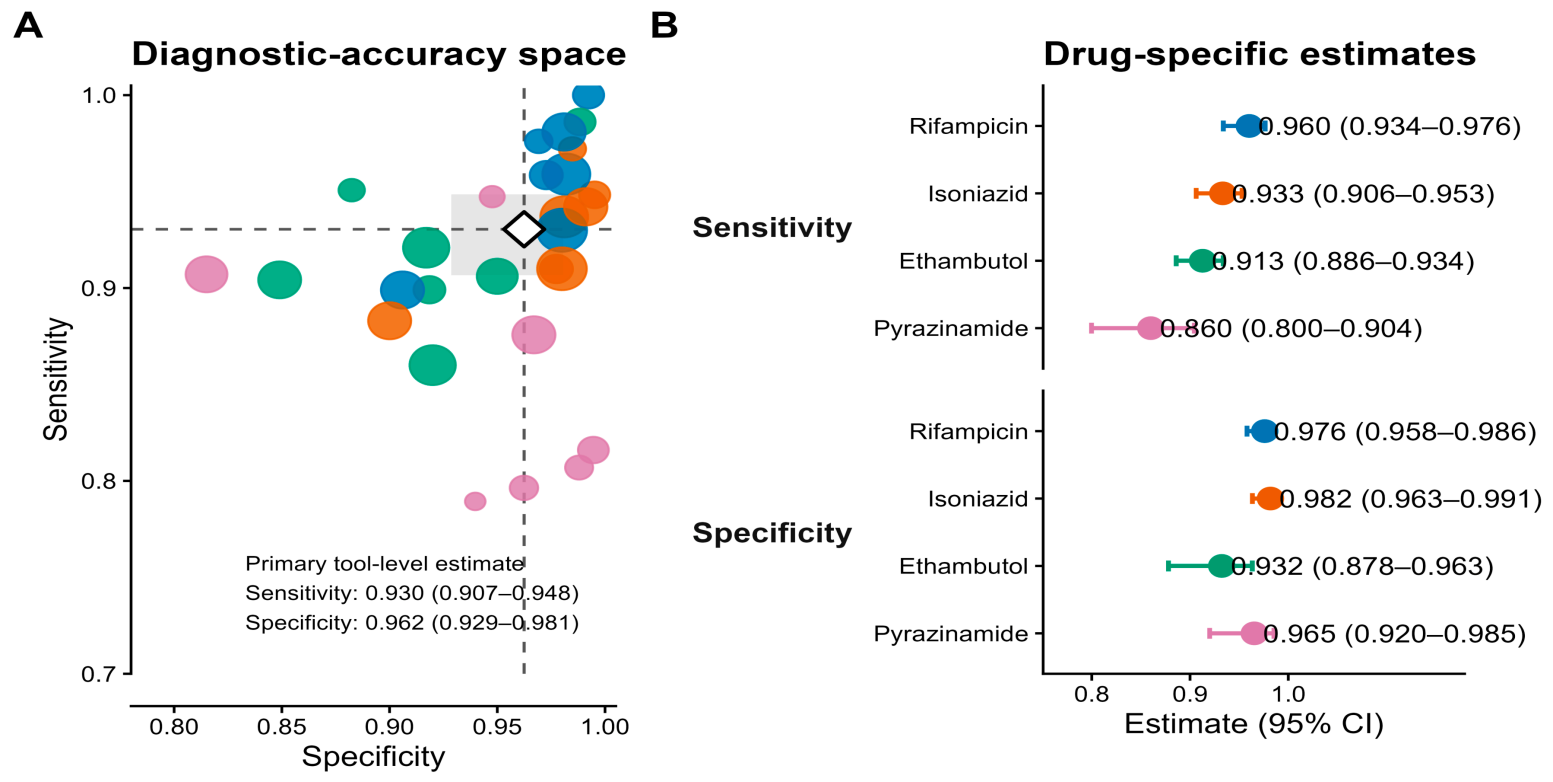
Tool-level and drug-specific diagnostic accuracy of WGS-based drug-resistance prediction. (**A**) Diagnostic-accuracy space for 28 drug-level records. Points are coloured by drug (rifampicin, blue; isoniazid, orange; ethambutol, green; pyrazinamide, pink) and sized by the number of isolates. The diamond represents the primary tool-level bivariate pooled estimate, and the shaded rectangle denotes its marginal 95% CI limits (sensitivity 0.930, 95% CI: 0.907–0.948; specificity 0.962, 95% CI: 0.929–0.981). The 28 drug-level records are displayed for context and were not treated as independent studies in the primary model. (**B**) Secondary drug-specific univariate random-effects estimates, each based on seven tools.

WGS-based resistance prediction demonstrated high overall first-line composite diagnostic accuracy. In the primary bivariate model fitted to seven tool-level composite 2 × 2 tables, pooled sensitivity was 0.930 (95% CI: 0.907–0.948) and pooled specificity was 0.962 (95% CI: 0.929–0.981) (Figure 2). The 28 drug-level records are displayed individually in Figure 2 but were not treated as independent studies in the primary model.

### 3.3. Drug-Specific Diagnostic Accuracy

In secondary drug-specific univariate random-effects analyses, each based on seven tools, sensitivity was highest for rifampicin (0.960, 95% CI: 0.934–0.976) and isoniazid (0.933, 95% CI: 0.906–0.953), intermediate for ethambutol (0.913, 95% CI: 0.886–0.934), and lowest for pyrazinamide (0.860, 95% CI: 0.800–0.904), consistent with recognised genotype–phenotype correlation challenges for this agent (Table 2). Specificity remained high across all four drugs (0.93–0.98). Second-line estimates are described narratively only and are not included in the quantitative synthesis pending per-drug data verification.

### 3.4. Subgroup Analyses by Algorithm Type

**Figure 3:**
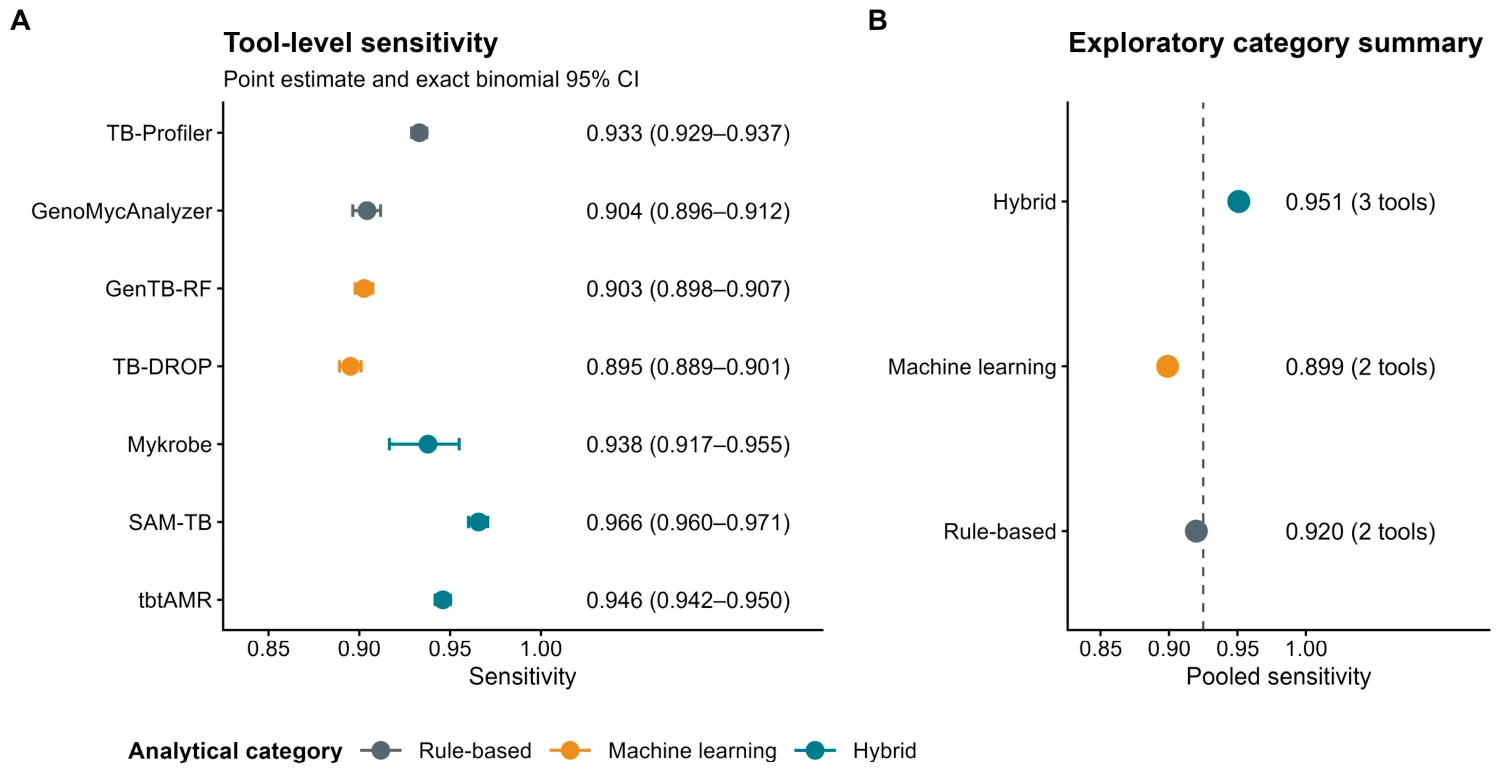
Forest plot of pooled sensitivity stratified by algorithm type. Panel (**A**) shows tool-level sensitivity with exact binomial 95% confidence intervals; panel (**B**) shows the exploratory category-level summary. Points are coloured by analytical category: rule-based (grey), machine learning (orange), and hybrid (teal). Pooled sensitivities: rule-based 0.920 (2 tools), machine learning 0.899 (2 tools), hybrid 0.951 (3 tools).

Because the four drug-specific evaluations contributed by each tool are not statistically independent, the exploratory comparison was conducted at the tool level by summing each tool’s four drug-specific 2 × 2 tables and fitting a bivariate random-effects model. Pooled sensitivity was 0.951 for hybrid tools, 0.920 for rule-based tools, and 0.899 for machine learning tools. The hybrid-versus-rule-based contrast was 0.533 on the logit scale (95% CI: 0.049–1.018; *p* = 0.031); the machine-learning-versus-rule-based contrast was −0.256 (95% CI: −0.773 to 0.262; *p* = 0.333). A sensitivity analysis using separate univariate models on the same seven tool-level units yielded *p* = 0.020 for the hybrid-versus-rule-based contrast and *p* = 0.311 for the machine-learning-versus-rule-based contrast. These estimates are descriptive and hypothesis-generating only: algorithm category is completely confounded with the identity, training data, mutation catalogue version, and validation population of the seven individual tools, and residual heterogeneity remained high. Tool- and category-level pooled sensitivity estimates are summarised in Figure 3, and a comparative overview of these tools, including their algorithm category and key reference, is provided in Table 3.

### 3.5. Meta-Regression Analysis

Because the 28 drug-level evaluations are nested within only seven tools, the exploratory comparison by algorithm category was conducted on the tool-level analytic unit. In the bivariate model, the hybrid-versus-rule-based contrast was 0.533 on the logit scale (95% CI: 0.049–1.018; *p* = 0.031), whereas the machine-learning-versus-rule-based contrast was −0.256 (95% CI: −0.773 to 0.262; *p* = 0.333). Separate univariate sensitivity models yielded *p* = 0.020 and *p* = 0.311, respectively. These results do not establish an algorithm-type effect: with only seven tools, algorithm category is completely confounded with individual tool identity, training data composition, mutation catalogue version, and validation population. The comparison is therefore reported as descriptive and hypothesis-generating; it should be evaluated in prospectively designed head-to-head studies using shared isolate collections.

### 3.6. Publication Bias

**Figure 4:**
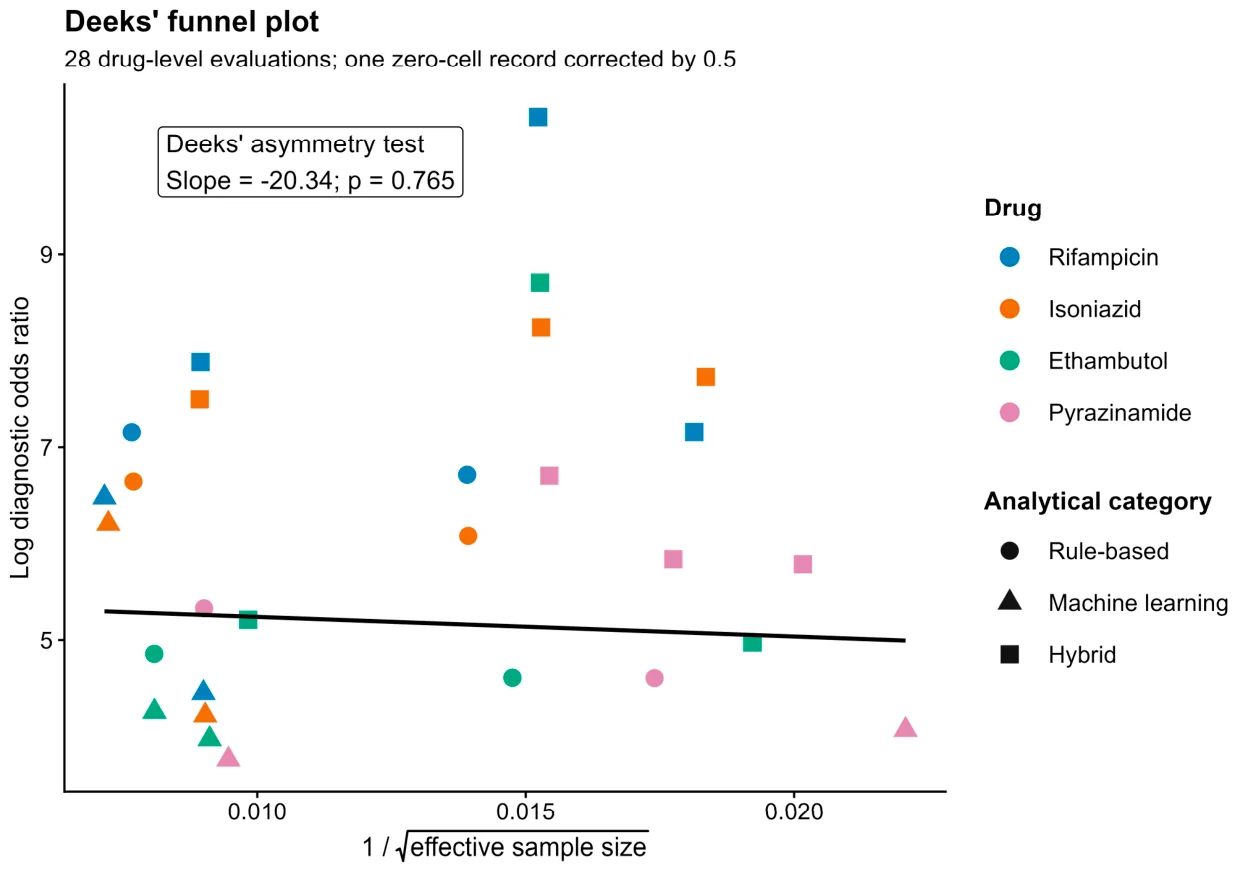
Exploratory Deeks’ funnel plot asymmetry test across 28 drug-level evaluations nested within 7 tools. Each point represents one tool-drug combination. Points are coloured by drug (rifampicin: blue; isoniazid: orange; ethambutol: green; pyrazinamide: pink) and shaped by analytical category (circle: rule-based; triangle: machine learning; square: hybrid). The fitted regression line shows no statistically significant asymmetry. Given the small number of independent tools and inherent clustering by tool, interpretation should be cautious.

Using the drug-level evaluations, an exploratory Deeks’ funnel plot asymmetry test showed a slope coefficient of −20.34 (*p* = 0.765), indicating no statistically significant evidence of publication bias (Figure 4). With only seven independent tools contributing 28 non-independent drug-level evaluations, this test has very limited statistical power, and a non-significant result should not be interpreted as evidence of an absence of publication bias. One dataset (Mykrobe rifampicin, FN = 0) required a 0.5, applied only to that row.

## 4. Discussion

This systematic review and meta-analysis found high overall first-line composite diagnostic accuracy at the tool level. In the primary bivariate analysis of seven tool-level composite tables, pooled sensitivity was 0.930 (95% CI: 0.907–0.948) and pooled specificity was 0.962 (95% CI: 0.929–0.981). The 28 drug-level evaluations were not treated as independent studies because each tool contributed four drug-specific records. The primary results are broadly consistent with previous systematic reviews [3,20,33]. Exploratory tool-level comparisons showed different sensitivity estimates across the three algorithm categories, but these comparisons cannot identify the contribution of algorithmic design because category is completely confounded with the characteristics of the individual tools and their source data.

The observed sensitivity gradient across drugs is consistent with known differences in the genetic architecture of resistance, although this ecological comparison cannot establish mechanism. Rifampicin resistance is overwhelmingly conferred by mutations within an 81-base pair hotspot in *rpoB* (codons 411–430), accounting for >95% of resistant isolates globally; this constrained mutational space is well-catalogued and yields highly predictable genotype–phenotype correlations [34]. Isoniazid resistance similarly concentrates in *katG* and the *inhA* regulatory region, supporting reliable genomic prediction.

In contrast, pyrazinamide resistance is characterised by profound genetic heterogeneity: causative *pncA* mutations are distributed across the full gene length, encompass more than 100 distinct variants in epidemiological surveys, and lack well-defined functional hotspots [35]. Accessory resistance-associated loci, including *rpoA* and *clpC1*, have been implicated but remain mechanistically incompletely characterised. These genetic complexities are compounded by phenotypic testing artefacts: pyrazinamide pDST results are exquisitely sensitive to medium pH, inoculum density, and incubation conditions, and demonstrate poor inter-laboratory reproducibility [36,37]. Accordingly, apparent WGS–pDST discordance for pyrazinamide likely reflects reference standard limitations rather than true tool failure; performance conclusions for this agent should be interpreted with appropriate caution.

Second-line drug resistance presents comparable mechanistic complexity. Fluoroquinolone resistance is mediated primarily by mutations in *gyrA* and *gyrB*, while bedaquiline and linezolid resistance [38] involve multiple loci (*atpE*, *pepQ*, *mmpR5*, *rrl*, *rplC*) with variable penetrance and lineage-dependent effects. Current mutation catalogues explain substantially fewer second-line than first-line resistance phenotypes, directly constraining the maximum achievable sensitivity for these drug classes. The WHO mutation catalogue is an important interpretive resource; however, the performance of catalogue-based WGS prediction remains variable for several second-line drugs and across settings [39].

The exploratory differences in pooled sensitivity across the algorithm categories should not be interpreted as evidence that algorithm type determines diagnostic performance. Rule-based tools use curated mutation catalogues and can offer transparent, interpretable resistance calls, especially for well-characterised first-line resistance mechanisms. However, the present data cannot separate any contribution of this design from differences in the tools, training data, catalogue versions, validation populations, or primary-study methods.

Machine learning tools showed a pooled sensitivity of 0.899 at the tool level, not significantly different from rule-based tools (*p* = 0.333); this is consistent with the reviewer-identified possibility that the originally reported gap partly reflected differing drug panels and within-tool pseudo-replication across algorithm categories rather than a genuine algorithm-type effect. Gröschel et al. demonstrated that even models trained on >20,000 isolates show marked performance degradation when applied to genetically distant populations, highlighting the fundamental dependence of data-driven methods on training set representativeness [14].

Extant training datasets are disproportionately enriched for Euro-American (lineage 4) and East Asian (lineage 2) strains, with systematic underrepresentation of African lineages (5 and 6) and South American isolates, consistent with documented global phylogeographic patterns [40]. Machine learning methodologies nevertheless retain substantial theoretical advantages—particularly for identifying epistatic and higher-order resistance determinants—and investment in globally representative training data coupled with rigorous prospective validation may substantially reduce the current sensitivity gap.

Hybrid tools (Mykrobe [13], tbtAMR, and SAM-TB) had the highest pooled sensitivity estimate among the three categories (0.951 at the tool level). The hybrid-versus-rule-based contrast reached *p* = 0.031 in the seven-tool bivariate analysis and *p* = 0.020 in a univariate sensitivity analysis. These results remain exploratory: only three hybrid and two rule-based tools contributed, and algorithm category is completely confounded with the identity of individual tools. Prospective head-to-head evaluations across diverse drug panels, *Mycobacterium tuberculosis* lineages, and geographic settings using identical isolate collections are needed before these observations inform tool selection, consistent with previously articulated calls for a more rigorous TB diagnostics research agenda [41].

The predictive accuracy of any WGS-based tool is fundamentally circumscribed by the completeness and global representativeness of the underlying genotype–phenotype evidence base. Geographic representation in existing datasets is heavily skewed toward Euro-American and East Asian lineages, and catalogue coverage is substantially higher for first-line than second-line agents. This systematic bias may compromise tool generalisability in high-burden African and South Asian settings, with direct implications for diagnostic equity and clinical outcomes. Sustained international investment in expanding globally representative datasets—building upon initiatives such as the CRyPTIC Consortium and the WHO TB Supranational Reference Laboratory Network—is essential for achieving equitable, high-performance WGS-based diagnostics across all high-burden settings.

The overall tool-level composite WGS specificity was 0.962 (95% CI: 0.929–0.981) supports the potential clinical value of WGS-based resistance prediction, particularly when resistance-associated variants are detected within well-characterised genomic regions. Nevertheless, turnaround time and clinical utility depend on whether sequencing is performed directly from clinical specimens or from cultured isolates, as well as on local laboratory infrastructure, sequencing workflow, bioinformatic capacity, and reporting procedures. However, the lower sensitivity for pyrazinamide and second-line agents indicates that a negative genomic prediction should not invariably be interpreted as evidence of phenotypic susceptibility. Negative WGS predictions for second-line agents—particularly in settings with elevated fluoroquinolone resistance—warrant cautious clinical interpretation and should prompt confirmatory pDST where feasible. At present, WGS may complement rather than universally replace pDST, with the appropriate combination of methods determined by drug, specimen type, local prevalence, and laboratory capacity. At the population level, standardised WGS surveillance platforms offer transformative potential for tracking the emergence and spread of drug-resistant strains, delineating transmission networks, and informing national TB control policies [20,28,42,43].

### Limitations

Several limitations merit explicit acknowledgement. Substantial heterogeneity remained after the exploratory comparison, indicating important unmeasured covariates, including mutation catalogue version and completeness, sequencing platform, variant-calling pipeline, pDST protocol, and characteristics of the validation population. Of the 19 included studies, seven contributed extractable contingency data to the quantitative synthesis; the 28 drug-level evaluations were clustered within seven tools and should not be interpreted as independent studies. The historical database search strings, search dates, original AI prompt, model version/date, and record-level screening audit trail were not retained in a recoverable working file. Reconstructed search strategies are provided in Supplementary File S1, but rerunning them may not reproduce the historical record counts. All AI-flagged exclusions were re-examined by two human reviewers, although individual decisions and any reinstatements were not retained. The quantitative synthesis is restricted to four first-line drugs. Supplementary Table S1 transparently classifies the data provenance for each 2 × 2 record; six records are provisional/reconstructed, and the corresponding sensitivity analysis is reported in Supplementary File S3. Finally, the algorithm category comparison is descriptive and hypothesis-generating because only two to three tools contributed to each category and category is completely confounded with individual tool identity and related characteristics. A formal GRADE certainty-of-evidence assessment was not conducted.

WGS-based bioinformatic tools provided a pooled tool-level composite specificity of 0.962 (95% CI: 0.929–0.981) and sensitivity of 0.930 (95% CI: 0.907–0.948) in *Mycobacterium tuberculosis* for first-line drug-resistance prediction across diverse clinical settings. WGS may complement phenotypic DST, with implementation guided by the drug, specimen type, local prevalence, laboratory capacity, and the need for confirmatory testing where genomic prediction is uncertain, consistent with current WHO diagnostic guidance [44].

## Figures and Tables

**Table 1:** Characteristics of the 19 studies included in the systematic review.

Study	Year	Region	N (Isolates)	Algorithm type	Tool/Catalogue	Included in MA
Phelan et al. [12]	2019	Multi-country	>17,000	Rule-based	TB-Profiler; WHO-based catalogue	Yes *
Gröschel et al. [14]	2021	Multi-country	20,408	Machine learning	GenTB (RF, WDNN); model-based	Yes *
Hunt et al. [22]	2019	Multi-country	10,207	Hybrid	Mykrobe; curated catalogue	Yes *
Yang et al. [15]	2022	China	3177	Hybrid	SAM-TB; integrated catalogue	Yes *
Kim et al. [16]	2024	South Korea	5473	Rule-based	GenoMycAnalyzer; WHO-based catalogue	Yes *
Wang et al. [17]	2024	China	~12,000	Machine learning	TB-DROP; deep learning framework	Yes *
Horan et al. [18]	2025	Multi-country	>15,000	Hybrid	tbtAMR; WHO catalogue + data-driven	Yes *
Kim et al. [23]	2022	South Korea	37	Mixed	Multiple tools	No
Lam et al. [24]	2021	Australia	1107	Unclassified	WGS pipeline	No
Feuerriegel et al. [25]	2015	Germany	92	Rule-based	PhyResSE; SNP mutation library	No
CRyPTIC Consortium [26]	2022	Global	12,289	Reference dataset	WGS; CRyPTIC catalogue	No
WHO catalogue study [27]	2023	Global	36,385	Catalogue-based	WHO catalogue of mutations	No
Miotto et al. [28]	2017	Multi-country	>3000	Catalogue-based	Standardised interpretation framework	No
Walker et al. [8]	2015	UK	>1000	Unclassified	WGS	No
Bradley et al. [13]	2015	UK	>1500	Hybrid	Mykrobe predictor; curated catalogue	No
Coll et al. [29]	2015	Spain	>500	Unclassified	WGS	No
Muzondiwa et al. [30]	2020	South Africa	>300	Rule-based	Resistance Sniffer; SNP-clade model	No
Ezewudo et al. [31]	2018	USA	>2000	Rule-based/DB	TB database; resistance knowledge base	No
Zade et al. [32]	2022	India	100	Unclassified	WGS analysis	No

Studies contributing extractable contingency data to the quantitative meta-analysis (*n* = 7) are indicated by asterisk (*), each contributing 4 drug-level datasets (rifampicin, isoniazid, ethambutol, pyrazinamide). RIF = rifampicin; INH = isoniazid; EMB = ethambutol; PZA = pyrazinamide; MDR-TB = multidrug-resistant tuberculosis; DB = database.

**Table 2:** Drug-specific diagnostic accuracy of WGS-based resistance prediction.

Drug	Evidence Basis	Sensitivity (95% CI)	Specificity (95% CI)
Rifampicin	Pooled across all 7 tools (verified 2 × 2 data)	0.960 (0.934–0.976)	0.976 (0.958–0.986)
Isoniazid	Pooled across all 7 tools (verified 2 × 2 data)	0.933 (0.906–0.953)	0.982 (0.963–0.991)
Ethambutol	Pooled across all 7 tools (verified 2 × 2 data)	0.913 (0.886–0.934)	0.932 (0.878–0.963)
Pyrazinamide	Pooled across all 7 tools (verified 2 × 2 data)	0.860 (0.800–0.904)	0.965 (0.920–0.985)
Second-line agents	Original narrative range; pending per-tool re-verification (see Limitations)	~0.84	~0.98

CI = confidence interval; pDST = phenotypic drug susceptibility testing. Rifampicin, isoniazid, ethambutol, and pyrazinamide values are secondary drug-specific univariate random-effects estimates across seven tools per drug. The primary overall estimate was derived separately from seven tool-level composite 2 × 2 tables (see Supplementary Table S1). Second-line agent estimates reflect the original narrative-synthesis range pending per-tool data re-verification.

**Table 3:** Comparative overview of representative WGS-based bioinformatic tools.

Tool	Category	Key Reference
TB-Profiler (version not reported)	Rule-based	[12]
Mykrobe (v0.13.0)	Hybrid	[13,22]
GenTB (RF/WDNN) (version not reported)	Machine learning	[14]
GenoMycAnalyzer (version not reported)	Rule-based	[16]
SAM-TB (web-based; version not separately recorded)	Hybrid	[15]
tbtAMR (version not reported by the source study)	Hybrid (clinical-grade)	[18]
TB-DROP (version not reported)	Machine learning (deep learning architecture)	[17]
PhyResSE (version not reported)	Rule-based	[25]
Resistance Sniffer (version not reported)	Rule-based	[30]

Note: TB-DROP’s category is labelled “Machine learning (deep learning architecture)” for consistency with the three-category framework defined in Section 1.

## Data Availability

All primary data supporting the results of this study are derived from previously published studies that are publicly available; full references are provided in the reference list. The extracted 2 × 2 contingency data used in the meta-analysis are provided in Supplementary Table S1. The extracted drug-level contingency data and complete R analysis code have been deposited in Zenodo (https://doi.org/10.5281/zenodo.21523100), licenced under CC-BY 4.0.

## Supplementary Materials

The following supporting information can be downloaded at: https://www.mdpi.com/article/10.3390/microorganisms14081824/s1. Table S1 (2×2 contingency table raw data), Table S2 (stratified sensitivity analyses by algorithm type and drug category), Table S3 (characteristics of 19 included studies), File S1 (comprehensive search strategies for five databases and AI-based screening methodology), File S2 (PRISMA-DTA Checklist with 29 items completed), and File S3 (R 4.5.2 statistical code and analytical dataset). QUADAS2_Assessment 8.1: QUADAS-2 risk-of-bias and applicability assessment workbook for all 19 included studies (domain-level and overall reviewer judgements and consensus, as referenced in Section 2.5); PRISMA_2020_Checklist: Completed PRISMA 2020 checklist for this systematic review and meta-analysis.

## References

[1] World Health Organization (2025). Global Tuberculosis Report 2025.

[2] Uplekar M., Weil D., Lonnroth K., Jaramillo E., Lienhardt C., Dias H.M., Falzon D., Floyd K., Gargioni G., Getahun H. (2015). WHO’s new End TB Strategy. Lancet.

[3] Chen Y.L., He Y., Dahl V.N., Yu K., Zhang Y.A., Guan C.P., Wang M.S. (2025). Diagnostic yield of nine user-friendly bioinformatics tools for predicting *Mycobacterium tuberculosis* drug resistance: A systematic review and network meta-analysis. PLoS Glob. Public Health.

[4] CRyPTICConsortium (2018). 100,000 Genomes Project. Prediction of susceptibility to first-line tuberculosis drugs by DNA sequencing. N. Engl. J. Med..

[5] Doyle R.M., Burgess C., Williams R., Gorton R., Booth H., Brown J., Bryant J.M., Chan J., Creer D., Holdstock J. (2018). Direct whole-genome sequencing of sputum accurately identifies drug-resistant *Mycobacterium tuberculosis* faster than MGIT culture sequencing. J. Clin. Microbiol..

[6] Gröschel M.I., Walker T.M., van der Werf T.S., Lange C., Niemann S., Merker M. (2018). Pathogen-based precision medicine for drug-resistant tuberculosis. PLoS Pathog..

[7] Farhat M.R., Shapiro B.J., Kieser K.J., Sultana R., Jacobson K.R., Victor T.C., Warren R.M., Streicher E.M., Calver A., Sloutsky A. (2013). Genomic analysis identifies targets of convergent positive selection in drug-resistant *Mycobacterium tuberculosis*. Nat. Genet..

[8] Walker T.M., Kohl T.A., Omar S.V., Hedge J., Del Ojo Elias C., Bradley P., Iqbal Z., Feuerriegel S., Niehaus K.E., Wilson D.J. (2015). Whole-genome sequencing for prediction of *Mycobacterium tuberculosis* drug susceptibility and resistance: A retrospective cohort study. Lancet Infect. Dis..

[9] Walker T.M., Fowler P.W., Knaggs J., Iqbal Z., Hunt M., Chindelevitch L., Farhat M., Cirillo D.M., CRyPTIC Consortium, the Seq&Treat Consortium (2022). The 2021 WHO catalogue of *Mycobacterium tuberculosis* complex mutations associated with drug resistance: A genotypic analysis. Lancet Microbe.

[10] World Health Organization (2021). Catalogue of Mutations in Mycobacterium tuberculosis Complex and Their Association with Drug Resistance.

[11] Witney A.A., Bateson A.L.E., Jindani A., Phillips P.P.J., Coleman D., Stoker N.G., Butcher P.D., McHugh T.D., RIFAQUIN Study Team (2017). Use of whole-genome sequencing to distinguish relapse from reinfection in a completed tuberculosis clinical trial. BMC Med..

[12] Phelan J.E., O’Sullivan D.M., Machado D., Ramos J., Oppong Y.E.A., Campino S., O’Grady J., McNerney R., Hibberd M.L., Viveiros M. (2019). Integrating informatics tools and portable sequencing technology for rapid detection of resistance to anti-tuberculosis drugs. Genome Med..

[13] Bradley P., Gordon N.C., Walker T.M., Dunn L., Heys S., Huang B., Earle S., Pankhurst L.J., Anson L., de Cesare M. (2015). Rapid antibiotic-resistance predictions from genome sequence data for *Staphylococcus aureus* and *Mycobacterium tuberculosis*. Nat. Commun..

[14] Gröschel M.I., Owens M., Freschi L., Vargas R., Marin M.G., Phelan J., Iqbal Z., Dixit A., Farhat M.R. (2021). GenTB: A user-friendly genome-based predictor for tuberculosis resistance powered by machine learning. Genome Med..

[15] Yang T., Gan M., Liu Q., Liang W., Tang Q., Luo G., Zuo T., Guo Y., Hong C., Li Q. (2022). SAM-TB: A whole genome sequencing data analysis website for detection of *Mycobacterium tuberculosis* drug resistance and transmission. Brief. Bioinform..

[16] Kim D., Shin J.I., Yoo I.Y., Jo S., Chu J., Cho W.Y., Shin S.H., Chung Y.J., Park Y.J., Jung S.H. (2024). GenoMycAnalyzer: A web-based tool for species and drug resistance prediction for *Mycobacterium* genomes. BMC Genom..

[17] Wang Y., Jiang Z., Liang P., Liu Z., Cai H., Sun Q. (2024). TB-DROP: Deep learning-based drug resistance prediction of *Mycobacterium tuberculosis* utilizing whole genome mutations. BMC Genom..

[18] Horan K.A., Viberg L., Ballard S.A., Globan M., Wirth W., Bond K., Webb J.R., Dorji T., Williamson D.A., Sait M.L. (2025). Bringing tuberculosis genomics to the clinic: Development and validation of a comprehensive pipeline to predict antimicrobial susceptibility from genomic data, accredited to ISO standards. Lancet Digit. Health.

[19] Nimmo C., Shaw L.P., Doyle R., Williams R., Brien K., Burgess C., Breuer J., Balloux F., Pym A.S. (2019). Whole genome sequencing *Mycobacterium tuberculosis* directly from sputum identifies more genetic diversity than sequencing from culture. BMC Genom..

[20] Tagami Y., Horita N., Kaneko M., Kaneko T. (2024). Whole-genome sequencing predicts phenotypic drug resistance in *Mycobacterium tuberculosis*: A systematic review and meta-analysis. J. Infect. Dis..

[21] McInnes M.D.F., Moher D., Thombs B.D., McGrath T.A., Bossuyt P.M., the PRISMA-DTA Group (2018). Preferred Reporting Items for a Systematic Review and Meta-analysis of Diagnostic Test Accuracy Studies: The PRISMA-DTA Statement. JAMA.

[22] Hunt M., Bradley P., Lapierre S.G., Heys S., Thomsit M., Hall M.B., Malone K.M., Wintringer P., Walker T.M., Cirillo D.M. (2019). Antibiotic resistance prediction for *Mycobacterium tuberculosis* from genome sequence data with Mykrobe. Wellcome Open Res..

[23] Kim N., Seok K.H., Lim H.-J., Kim S.-Y., Cho S.-B., Choi Y., Song S.H., Lee J.H. (2022). Evaluation of Five User-Friendly Whole Genome Sequencing Software for *Mycobacterium tuberculosis* in Clinical Application. J. Korean Med. Sci..

[24] Lam C., Martinez E., Crighton T., Furlong C., Donnan E., Marais B.J., Sintchenko V. (2021). Value of routine whole genome sequencing for *Mycobacterium tuberculosis* drug resistance detection. Int. J. Infect. Dis..

[25] Feuerriegel S., Schleusener V., Beckert P., Kohl T.A., Miotto P., Cirillo D.M., Cabibbe A.M., Niemann S., Fellenberg K. (2015). PhyResSE: A web tool delineating *Mycobacterium tuberculosis* antibiotic resistance and lineage from whole-genome sequencing data. J. Clin. Microbiol..

[26] The CRyPTIC Consortium (2022). A data compendium associating the genomes of 12,289 *Mycobacterium tuberculosis* isolates with quantitative resistance phenotypes to 13 antibiotics. PLoS Biol..

[27] Chen Y., Zhang X., Liang J., Jiang Q., Peierdun M., Xu P., Takiff H.E., Gao Q. (2025). Advantages of updated WHO mutation catalog combined with existing whole-genome sequencing-based approaches for *Mycobacterium tuberculosis* resistance prediction. Genome Med..

[28] Miotto P., Tessema B., Tagliani E., Chindelevitch L., Starks A.M., Emerson C., Hanna D., Kim P.S., Liwski R., Zignol M. (2017). A standardised method for interpreting the association between mutations and phenotypic drug resistance in *Mycobacterium tuberculosis*. Eur. Respir. J..

[29] Coll F., McNerney R., Preston M.D., Guerra-Assunção J.A., Warry A., Hill-Cawthorne G., Mallard K., Nair M., Miranda A., Alves A. (2015). Rapid determination of anti-tuberculosis drug resistance from whole-genome sequences. Genome Med..

[30] Muzondiwa D., Mutshembele A., Pierneef R.E., Reva O.N. (2020). Resistance Sniffer: An online tool for prediction of drug resistance patterns of *Mycobacterium tuberculosis* isolates using next generation sequencing data. Int. J. Med. Microbiol..

[31] Ezewudo M., Borens A., Chiner-Oms Á., Miotto P., Chindelevitch L., Starks A.M., Hanna D., Liwski R., Zignol M., Gilpin C. (2018). Integrating standardized whole genome sequence analysis with a global *Mycobacterium tuberculosis* antibiotic resistance knowledgebase. Sci. Rep..

[32] Zade A., Shah S., Hirani N., Kondabagil K., Joshi A., Chatterjee A. (2022). Whole-genome sequencing of presumptive MDR-TB isolates from a tertiary healthcare setting in Mumbai. J. Glob. Antimicrob. Resist..

[33] Perea-Jacobo R., Paredes-Gutiérrez G.R., Guerrero-Chevannier M.Á., Flores D.-L., Muñiz-Salazar R. (2023). Machine Learning of the Whole Genome Sequence of *Mycobacterium tuberculosis*: A Scoping PRISMA-Based Review. Microorganisms.

[34] Schön T., Miotto P., Köser C.U., Viveiros M., Böttger E., Cambau E. (2017). *Mycobacterium tuberculosis* drug-susceptibility testing: Challenges and opportunities in the era of whole-genome sequencing. Clin. Microbiol. Infect..

[35] Yadon A.N., Maharaj K., Adamson J.H., Lai Y.P., Sacchettini J.C., Ioerger T.R., Rubin E.J., Pym A.S. (2017). A comprehensive characterization of PncA polymorphisms that confer resistance to pyrazinamide. Nat. Commun..

[36] Roengsanthia D., Sangmanee A., Sangthong S., Angkasekwinai N., Muangnoicharoen S. (2020). Discordance between phenotypic and genotypic drug susceptibility testing in *Mycobacterium tuberculosis*: A systematic analysis. Antibiotics.

[37] Whitfield M.G., Warren R.M., Streicher E.M., Sampson S.L., Sirgel F.A., van Helden P.D., Mercante A., Willby M., Hughes K., Birkness K. (2015). *Mycobacterium tuberculosis pncA* polymorphisms that do not confer pyrazinamide resistance at a breakpoint concentration of 100 µg/mL in MGIT. J. Clin. Microbiol..

[38] Nguyen T.V.A., Anthony R.M., Bañuls A.L., Nguyen T.V.A., Vu D.H., Alffenaar J.W.C. (2018). Bedaquiline resistance: Its emergence, mechanism, and prevention. Clin. Infect. Dis..

[39] He G., Zheng Q., Shi J., Wu L., Huang B., Yang Y. (2024). Evaluation of WHO catalog of mutations and five WGS analysis tools for drug resistance prediction of *Mycobacterium tuberculosis* isolates from China. Microbiol. Spectr..

[40] Freschi L., Vargas R., Hussain A., Kamal S.M.M., Skrahina A., Tahseen S., Ismail N., Barbova A., Niemann S., Cirillo D.M. (2021). Population structure, biogeography and transmissibility of *Mycobacterium tuberculosis*. Nat. Commun..

[41] Pai M., Schumacher S.G., Lienhardt C. (2016). Sitting with discomfort—A research agenda to guide the use of diagnostic tests for tuberculosis. Lancet Infect. Dis..

[42] Ektefaie Y., Dixit A., Freschi L., Farhat M.R. (2021). Globally diverse *Mycobacterium tuberculosis* resistance acquisition: A retrospective geographical and temporal analysis of whole genome sequences. Lancet Microbe.

[43] Zignol M., Cabibbe A.M., Dean A.S., Glaziou P., Alikhanova N., Ama C., Andres S., Barbova A., Borbe-Reyes A., Chin D.P. (2018). Genetic sequencing for surveillance of drug resistance in tuberculosis in highly endemic countries: A multi-country population-based surveillance study. Lancet Infect. Dis..

[44] World Health Organization (2023). WHO Consolidated Guidelines on Tuberculosis: Module 3—Diagnosis.

