# Supplementary material for: Algorithmic Determinants of Performance Heterogeneity in Whole-Genome Sequencing-Based Prediction of Drug Resistance in Mycobacterium tuberculosis: A Systematic Review and Meta-Analysis
Source: Microorganisms. 2026 Aug 18;14(8):1824. doi: 10.3390/microorganisms14081824 (PMC13515934; doi:10.3390/microorganisms14081824)
Supplement: Supplementary file 1 [file microorganisms-14-01824-s001.zip › PRISMA_2020_Checklist.pdf]

PRISMA 2020 CHECKLIST

Algorithmic Determinants of Performance Heterogeneity in Whole-Genome Sequencing-Based Prediction of Drug Resistance in Mycobacterium tuberculosis: A Systematic Review and Meta-analysis

Section/Topic	#	Checklist item	Location where item is reported
TITLE			
Title	1	Identify the report as a systematic review.	Title page: '...A Systematic Review and Meta-Analysis.'
ABSTRACT			
Abstract	2	See the PRISMA 2020 for Abstracts checklist.	Abstract (Background/Methods/Results/Conclusions structure)
INTRODUCTION			
Rationale	3	Describe the rationale for the review in the context of existing knowledge.	Introduction, paragraphs 1-5
Objectives	4	Provide an explicit statement of the objective(s) or question(s).	Introduction, final paragraph ('We therefore conducted...')
METHODS			
Eligibility criteria	5	Specify inclusion/exclusion criteria and how studies were grouped for synthesis.	Methods 2.3
Information sources	6	Specify all databases, registers, etc., and last-search dates.	Methods 2.2; Supplementary File S1
Search strategy	7	Present full search strategies for all databases.	Supplementary File S1. The exact historical search strings and dates were not retained; reconstructed strategies are provided and disclosed as such (see Limitations); the historical record counts in Figure 1 are confirmed by the authors as accurate contemporaneous results
Selection process	8	Methods to decide study inclusion; number/independence of reviewers; automation tools.	Methods 2.2-2.3. AI-assisted prescreening (DeepSeek) was used as a decision-support tool only; all AI-flagged exclusions were re-examined by two human reviewers. A record-level audit trail of individual reviewer decisions and any reinstatements was not retained (disclosed in Methods 2.2 and Limitations)
Data collection process	9	Methods to collect data; number/independence of reviewers; automation tools.	Methods 2.4
Data items	10a	List/define outcomes sought.	Methods 2.4 (2x2 contingency data: TP/FP/FN/TN)
Data items	10b	List/define other variables sought.	Methods 2.4; Supplementary Table S3 (detailed full-text characteristics are verified for the seven quantitative studies; stated data gaps remain for the other 12 included studies).

Study risk of bias assessment	11	Methods to assess risk of bias; tool(s); reviewers; automation.	Methods 2.5 (QUADAS-2, independently assessed by two reviewers for all 19 included studies; disagreements resolved by consensus)
Effect measures	12	Effect measure(s) used.	Methods 2.6 (sensitivity, specificity, logit transformation)
Synthesis methods	13a	Methods to decide which studies were eligible for each synthesis.	Methods 2.3, 2.6
Synthesis methods	13b	Methods to prepare data (missing statistics, conversions).	Methods 2.4, 2.6; Supplementary Table S1
Synthesis methods	13c	Methods to tabulate/display individual study and synthesis results.	Table 1, Table 2; Figures 2-3
Synthesis methods	13d	Synthesis model(s)/method(s), heterogeneity assessment, software.	Methods 2.6 (metafor::rma.mv, R 4.3.3)
Synthesis methods	13e	Methods to explore heterogeneity (subgroup, meta-regression).	Methods 2.6; Results 3.4-3.5
Synthesis methods	13f	Sensitivity analyses conducted.	Methods 2.6; Results 3.4; Supplementary File S3. Includes leave-one-tool-out analysis and a sensitivity analysis excluding the 6 lower-confidence (Provisional/reconstructed) drug-level records
Reporting bias assessment	14	Methods to assess risk of bias due to missing results.	Methods 2.6 (Deeks' test); explicitly noted as low-powered and not interpreted as evidence for or against publication bias
Certainty assessment	15	Methods to assess certainty in the body of evidence.	NOT PERFORMED - a formal GRADE certainty-of-evidence assessment was not conducted in this review; this is an acknowledged limitation, disclosed in the Discussion (Limitations)
RESULTS			
Study selection	16a	Results of search/selection, ideally with flow diagram.	Results 3.1; Figure 1 (PRISMA flow diagram)
Study selection	16b	Cite and explain studies excluded that might appear eligible.	Results 3.1 (aggregate reasons and counts, consistent with PRISMA flow: 45+52+48+56+8=209). A study-level list of the 209 excluded reports is not provided as a supplementary table in this submission.
Study characteristics	17	Cite each included study and its characteristics.	Table 1; Supplementary Table S3 (detailed full-text characteristics are verified for the seven quantitative studies; stated data gaps remain for the other 12 included studies).
Risk of bias in studies	18	Present risk-of-bias assessments for each included study.	Updated QUADAS-2 workbook. QUADAS-2 was independently assessed by two reviewers for all 19 included studies; disagreements were resolved by consensus. Domain-level and reviewer-level assessments, including consensus judgements, are provided in the workbook.

Results of individual studies	19	Summary statistics and effect estimates for each study.	Supplementary Table S1 (28 drug-level 2x2 tables, with data provenance: 8 Direct, 14 Reconstructed, 6 Provisional/reconstructed); Supplementary Table S2
Results of syntheses	20a	Summarise characteristics/risk of bias among contributing studies.	Results 3.1; Discussion, Limitations
Results of syntheses	20b	Present synthesis results (summary estimate, precision, heterogeneity).	Results 3.2-3.4; Table 2; Figures 2-3
Results of syntheses	20c	Present results of heterogeneity investigations.	Results 3.5 (meta-regression, described as a descriptive, exploratory comparison across 7 tool-level analytic units)
Results of syntheses	20d	Present sensitivity analysis results.	Results 3.4; Supplementary File S3. Effect sizes and 95% confidence intervals for both the primary model and the lower-confidence-record exclusion analysis are reported
Reporting biases	21	Present risk-of-bias-due-to-missing-results assessments.	Results 3.6 (Deeks' test, with explicit low-power caveat)
Certainty of evidence	22	Present certainty assessments for each outcome.	NOT PERFORMED - see Item 15
DISCUSSION			
Discussion	23a	General interpretation of results in context of other evidence.	Discussion, paragraphs 1-2
Discussion	23b	Limitations of the evidence included.	Discussion, Limitations paragraph
Discussion	23c	Limitations of the review process.	Discussion, Limitations paragraph (search/AI-screening reconstruction disclosed; certainty assessment not performed)
Discussion	23d	Implications for practice, policy, and future research.	Discussion, concluding paragraphs
OTHER INFORMATION			
Registration and protocol	24a	Registration information (register name, number) or state not registered.	Methods 2.1: registered in PROSPERO (CRD420261342739)
Registration and protocol	24b	Where the protocol can be accessed.	Study protocol available on request; not deposited in a public registry beyond PROSPERO
Registration and protocol	24c	Amendments to registered information/protocol.	Discussion, Limitations (algorithm-type moderator analysis corrected post hoc to the level of 7 tool-level analytic units; drug-class and study-size moderators not repeated; disclosed as a deviation from the original analysis plan)
Support	25	Sources of financial/non-financial support; role of funders.	Funding section

Competing interests	26	Competing interests of review authors.	Conflicts of Interest section
Availability of data, code and other materials	27	Public availability of forms, extracted data, analytic data, code, other materials.	Data Availability Statement: data and R code deposited in Zenodo, DOI 10.5281/zenodo.21523100 (CC-BY 4.0); Supplementary Tables S1-S3; Supplementary Files S1, S2, S3
