# Supplementary material for: Algorithmic Determinants of Performance Heterogeneity in Whole-Genome Sequencing-Based Prediction of Drug Resistance in Mycobacterium tuberculosis: A Systematic Review and Meta-Analysis
Source: Microorganisms. 2026 Aug 18;14(8):1824. doi: 10.3390/microorganisms14081824 (PMC13515934; doi:10.3390/microorganisms14081824)
Supplement: Supplementary file 1 [file microorganisms-14-01824-s001.zip › Supplementary_File_S1.pdf]

SUPPLEMENTARY FILE S1 (Version 4)

Reconstructed Electronic Search Strategy and AI-Assisted Screening Protocol

Search period: 1 January 2000 to 28 January 2026 | Protocol version 1.0 | Reconstructed on 26 July 2026

1. Critical Database Clarification

The databases are reported as four sources, not five: MEDLINE via PubMed, Embase, Web of Science Core Collection, and Cochrane CENTRAL. PubMed is the search platform and contains MEDLINE plus additional records; "PubMed" and "PubMed (MEDLINE)" are not counted as two independent databases. Ovid MEDLINE was not searched separately in this review and is not reported as a fifth source.

Manuscript wording: "MEDLINE via PubMed, Embase, Web of Science Core Collection, and the Cochrane Central Register of Controlled Trials (CENTRAL) were searched from 1 January 2000 through 28 January 2026."

2. Status and Transparency Statement

These strategies are reconstructed from the manuscript, review question, named databases, eligibility criteria, included tools, and stated search dates. They are NOT the original historical search strings or the original AI prompt, because those records were not preserved in a recoverable working file. They are labelled 'reconstructed search strategies' throughout the manuscript, Response to Reviewers letter, and this supplementary file. These reconstructed strategies have not yet been executed against the live databases; the search histories, result counts, exported files, and screenshots that would constitute a new auditable source record have not been generated, and no claim of an executed rerun is made until that work is completed.

3. Search Design

- Concept A — condition: tuberculosis / Mycobacterium tuberculosis complex.
- Concept B — index method: whole-genome sequencing and genomic sequencing.
- Concept C — target condition and prediction framework: anti-tuberculosis drug resistance/susceptibility, phenotypic DST, genotype–phenotype prediction, bioinformatic tools, algorithms, and named tools.

No diagnostic-accuracy search filter (sensitivity/specificity/ROC/2×2 terminology) is applied at the search stage, since requiring this terminology in the search may miss eligible validation studies that report these data only in full text or supplements. No language, human, article-type, or full-text-availability filter is applied at the search stage; these criteria are handled during screening.

4. MEDLINE via PubMed

Run in PubMed Advanced Search. Export all results in .nbib format and CSV. Record the exact run date, result count, Search Details translation, and query URL.

("Tuberculosis"[Mesh] OR "Mycobacterium tuberculosis"[Mesh] OR tuberculo*[tiab] OR "Mycobacterium tuberculosis"[tiab] OR "M. tuberculosis"[tiab] OR MTB[tiab]) AND ("Whole Genome Sequencing"[Mesh] OR "whole genome sequencing"[tiab] OR "whole-genome sequencing"[tiab] OR WGS[tiab] OR "genome sequencing"[tiab] OR "genomic sequencing"[tiab]) AND ("Drug Resistance, Bacterial"[Mesh] OR "Microbial Sensitivity Tests"[Mesh] OR drug-resistan*[tiab] OR antimicrobial-resistan*[tiab] OR antibiotic-resistan*[tiab] OR susceptib*[tiab] OR "drug susceptibility"[tiab] OR "phenotypic drug susceptibility"[tiab] OR pDST[tiab] OR genotype-phenotype[tiab] OR "genotype phenotype"[tiab] OR "resistance prediction"[tiab] OR "resistance

detection"[tiab] OR "resistance classification"[tiab] OR "mutation catalogue"[tiab] OR "mutation catalog"[tiab] OR bioinformatic*[tiab] OR software[tiab] OR algorithm*[tiab] OR "machine learning"[tiab] OR "deep learning"[tiab] OR TB-Profiler[tiab] OR TBProfiler[tiab] OR Mykrobe[tiab] OR GenTB[tiab] OR "GenoMycAnalyzer"[tiab] OR "SAM-TB"[tiab] OR tbtAMR[tiab] OR "TB-DROP"[tiab] OR MTBseq[tiab] OR KvarQ[tiab] OR PhyResSE[tiab] OR CASTB[tiab]) AND (2000/01/01:2026/01/28[dp])

Records retrieved (once executed): [NOT YET EXECUTED — the reconstructed query above has not been run against the live database; a real record count, export file, and search date must be recorded here before this can be described as a completed rerun]

5. Optional Ovid MEDLINE Translation

Use only if the team genuinely searched, and intends to report, Ovid MEDLINE as a separate database. Both PubMed and Ovid MEDLINE are not counted as independent evidence sources without explaining overlap and deduplication.

1. exp Tuberculosis/ or exp Mycobacterium tuberculosis/ 2. (tuberculo* or Mycobacterium tuberculosis or M. tuberculosis or MTB).ti,ab,kf. 3. 1 or 2 4. exp Whole Genome Sequencing/ 5. (whole genome sequencing or whole-genome sequencing or WGS or genome sequencing or genomic sequencing).ti,ab,kf. 6. 4 or 5 7. exp Drug Resistance, Bacterial/ or exp Microbial Sensitivity Tests/ 8. (drug-resistan* or antimicrobial-resistan* or antibiotic-resistan* or susceptib* or drug susceptibility or phenotypic drug susceptibility or pDST or genotype-phenotype or genotype phenotype or resistance prediction or resistance detection or resistance classification or mutation catalogue or mutation catalog or bioinformatic* or software or algorithm* or machine learning or deep learning or TB-Profiler or TBProfiler or Mykrobe or GenTB or GenoMycAnalyzer or SAM-TB or tbtAMR or TB-DROP or MTBseq or KvarQ or PhyResSE or CASTB).ti,ab,kf. 9. 7 or 8 10. 3 and 6 and 9 11. limit 10 to yr="2000 - 2026" 12. limit 11 to date=20000101-20260128

6. Embase.com

Run in Embase Advanced Search. Keep Embase and MEDLINE source collections enabled only according to the prespecified plan; document explicitly whether MEDLINE records are included or excluded. Export RIS/CSV with Emtree terms and abstracts.

#1 'tuberculosis'/exp OR 'mycobacterium tuberculosis'/exp OR tuberculo*:ti,ab,kw OR 'mycobacterium tuberculosis':ti,ab,kw OR 'm. tuberculosis':ti,ab,kw OR mtb:ti,ab,kw #2 'whole genome sequencing'/exp OR 'whole genome sequencing':ti,ab,kw OR 'whole-genome sequencing':ti,ab,kw OR wgs:ti,ab,kw OR 'genome sequencing':ti,ab,kw OR 'genomic sequencing':ti,ab,kw #3 'bacterial drug resistance'/exp OR 'antimicrobial susceptibility test'/exp OR drug-resistan*:ti,ab,kw OR antimicrobial-resistan*:ti,ab,kw OR antibiotic-resistan*:ti,ab,kw OR susceptib*:ti,ab,kw OR 'drug susceptibility':ti,ab,kw OR 'phenotypic drug susceptibility':ti,ab,kw OR pdst:ti,ab,kw OR 'genotype phenotype':ti,ab,kw OR 'genotype-phenotype':ti,ab,kw OR 'resistance prediction':ti,ab,kw OR 'resistance detection':ti,ab,kw OR 'resistance classification':ti,ab,kw OR 'mutation catalogue':ti,ab,kw OR 'mutation catalog':ti,ab,kw OR bioinformatic*:ti,ab,kw OR software:ti,ab,kw OR algorithm*:ti,ab,kw OR 'machine learning':ti,ab,kw OR 'deep learning':ti,ab,kw OR 'tb-profiler':ti,ab,kw OR tbprofiler:ti,ab,kw OR mykrobe:ti,ab,kw OR gentb:ti,ab,kw OR genom*analyzer:ti,ab,kw OR 'sam-tb':ti,ab,kw OR tbtamr:ti,ab,kw OR 'tb-drop':ti,ab,kw OR mtbseq:ti,ab,kw OR kvarq:ti,ab,kw OR phyresse:ti,ab,kw OR castb:ti,ab,kw #4 #1 AND #2 AND #3 #5 #4 AND [2000-2026]/py #6 #5 AND [01-01-2000]/pd NOT [29-01-2026]/pd

Records retrieved (once executed): [NOT YET EXECUTED — the reconstructed query above has not been run against the live database; a real record count, export file, and search date must be recorded here before this can be described as a completed rerun]

7. Web of Science Core Collection

Run as an Advanced Search in Web of Science Core Collection. Keep all document types and all languages. Export full records and cited references in batches, recording indexes and exact timespan.

TS=(tuberculo* OR "Mycobacterium tuberculosis" OR "M. tuberculosis" OR MTB) AND TS=("whole genome sequencing" OR "whole-genome sequencing" OR WGS OR "genome sequencing" OR "genomic sequencing") AND TS=(drug-resistan* OR antimicrobial-resistan* OR antibiotic-resistan* OR susceptib* OR "drug susceptibility" OR "phenotypic drug susceptibility" OR

pDST OR "genotype-phenotype" OR "genotype phenotype" OR "resistance prediction" OR "resistance detection" OR "resistance classification" OR "mutation catalogue" OR "mutation catalog" OR bioinformatic* OR software OR algorithm* OR "machine learning" OR "deep learning" OR "TB-Profiler" OR TBProfiler OR Mykrobe OR GenTB OR GenoMycAnalyzer OR "SAM-TB" OR tbtAMR OR "TB-DROP" OR MTBseq OR KvarQ OR PhyResSE OR CASTB) Timespan: 2000-01-01 to 2026-01-28; Indexes: Web of Science Core Collection; Document types: All; Languages: All.

Records retrieved (once executed): [NOT YET EXECUTED — the reconstructed query above has not been run against the live database; a real record count, export file, and search date must be recorded here before this can be described as a completed rerun]

8. Cochrane CENTRAL

Run in Cochrane Library Search Manager and restrict the source to Trials/CENTRAL. Export citation records and record the date and result count.

#1 MeSH descriptor: [Tuberculosis] explode all trees #2 MeSH descriptor: [Mycobacterium tuberculosis] explode all trees #3 (tuberculo* OR "Mycobacterium tuberculosis" OR "M. tuberculosis" OR MTB):ti,ab,kw #4 #1 OR #2 OR #3 #5 MeSH descriptor: [Whole Genome Sequencing] explode all trees #6 ("whole genome sequencing" OR "whole-genome sequencing" OR WGS OR "genome sequencing" OR "genomic sequencing"):ti,ab,kw #7 #5 OR #6 #8 MeSH descriptor: [Drug Resistance, Bacterial] explode all trees #9 MeSH descriptor: [Microbial Sensitivity Tests] explode all trees #10 (drug-resistan* OR antimicrobial-resistan* OR antibiotic-resistan* OR susceptib* OR "drug susceptibility" OR "phenotypic drug susceptibility" OR pDST OR "genotype-phenotype" OR "genotype phenotype" OR "resistance prediction" OR "resistance detection" OR "resistance classification" OR "mutation catalogue" OR "mutation catalog" OR bioinformatic* OR software OR algorithm* OR "machine learning" OR "deep learning" OR "TB-Profiler" OR TBProfiler OR Mykrobe OR GenTB OR GenoMycAnalyzer OR "SAM-TB" OR tbtAMR OR "TB-DROP" OR MTBseq OR KvarQ OR PhyResSE OR CASTB):ti,ab,kw #11 #8 OR #9 OR #10 #12 #4 AND #7 AND #11 #13 #12 with Cochrane Library publication date between Jan 2000 and 28 Jan 2026

Records retrieved (once executed): [NOT YET EXECUTED — the reconstructed query above has not been run against the live database; a real record count, export file, and search date must be recorded here before this can be described as a completed rerun]

9. Limits and Database Settings

Setting	Required specification
Date	Publication date from 1 January 2000 through 28 January 2026, inclusive.
Language	No restriction.
Species	No automated human/animal filter. Exclude non-clinical or ineligible collections during full-text screening.
Document type	No automated restriction in the main search. Reviews, editorials, protocols, and abstracts without adequate data are excluded during screening but retained for citation chasing where useful.
Embase source setting	Record explicitly whether Embase-only or Embase+MEDLINE was searched. For a four-source strategy including PubMed, Embase-only is preferable to reduce duplicate MEDLINE retrieval, provided this is documented.
Web of Science indexes	Record the exact Core Collection indexes enabled (e.g., SCI-EXPANDED, ESCI, CPCI-S), since coverage depends on institutional subscription.
CENTRAL source	Trials/CENTRAL only; Cochrane Reviews are not counted as primary diagnostic-accuracy studies.
Search update	Because the intended cutoff is 28 January 2026, an update search from 29 January 2026 to the actual rerun date is run separately, retained as an update set, with a prospective decision on whether newly identified studies are incorporated before resubmission.

10. Search Execution and Deduplication Workflow

1. If and when these reconstructed strategies are executed, run each database search without modifying terms after the first database is exported; if a correction is necessary, version the strategy and re-execute all databases.

2. For each source, save: database/platform, institutional interface, exact query, limits, date/time, result count, screenshot/PDF of search history, and native export file.

3. Export PubMed as .nbib and CSV; Embase as RIS and CSV; Web of Science as plain text/RIS with full records and cited references; CENTRAL as RIS/CSV.

4. Import all files into one citation manager. Preserve a source-database field before deduplication.

5. Deduplicate in stages: exact DOI; PMID; exact title+year; normalized title+first author+year; then manual review of near-duplicates.

6. Retain a deduplication log showing the number removed at each stage. Do not delete companion reports until linkage is assessed.

7. Conduct backward and forward citation searching for all included studies and relevant systematic reviews; record these as "other methods," not as database search results.

8. Use two independent human reviewers for title/abstract and full-text screening. AI may prioritise or suggest decisions but must not replace independent human decisions.

11. Reconstructed AI-Assisted Screening Prompt

The following is a new, version-controlled reconstructed prompt. It is not described as the original prompt used historically, and has not itself been executed against a live AI screening run.

SYSTEMATIC REVIEW SCREENING PROMPT — RECONSTRUCTED VERSION 1.0

Role

You are assisting with citation screening for a PRISMA-DTA systematic review of whole-genome-sequencing-based prediction of drug resistance in Mycobacterium tuberculosis. You are not the final decision-maker. Two human reviewers must independently verify all inclusion and exclusion decisions.

Review question

Among clinical Mycobacterium tuberculosis isolates or specimens with a phenotypic drug-susceptibility-testing reference standard, what is the diagnostic accuracy of WGS-based bioinformatic tools, algorithms, mutation catalogues, or prediction frameworks for anti-tuberculosis drug resistance, and does performance differ by algorithm type?

Eligible study characteristics

1. Population/specimens: clinical M. tuberculosis complex isolates or direct clinical specimens from patients with suspected or confirmed tuberculosis.

2. Index test: whole-genome sequencing followed by an identifiable resistance-prediction tool, algorithm, pipeline, mutation catalogue, rule set, statistical model, machine-learning model, deep-learning model, or hybrid framework.

3. Target condition: resistance or susceptibility to one or more anti-tuberculosis drugs.

4. Reference standard: phenotypic drug susceptibility testing (pDST), MIC-based phenotyping, or another prespecified phenotypic reference standard.

5. Outcomes: sufficient information to extract or reconstruct TP, FP, FN, and TN, or at minimum sensitivity/specificity with denominators allowing reconstruction.

6. Study type: original diagnostic-accuracy, validation, benchmarking, cohort, cross-sectional, or retrospective/prospective clinical-isolate study.

7. Date: publication date from 1 January 2000 through 28 January 2026.

8. Language: no language restriction at search stage; flag non-English records for translation rather than excluding them automatically.

Exclude when clearly applicable

- E1. Not Mycobacterium tuberculosis complex.
- E2. No whole-genome sequencing index test.
- E3. No prediction of anti-tuberculosis drug resistance/susceptibility.
- E4. No phenotypic reference standard.
- E5. No extractable or reconstructable diagnostic 2×2 data after checking tables, supplements, and reported denominators.
- E6. Non-clinical or purely simulated/synthetic isolate collection with no eligible clinical validation, according to the review protocol.
- E7. Review, editorial, commentary, protocol, conference abstract without sufficient full data, guideline, or methods paper with no eligible validation dataset.
- E8. Duplicate or overlapping report: retain the most complete report, but record all companion reports and do not discard before human assessment.
- E9. Publication outside the prespecified date range.

Title/abstract screening instructions

For each record, output one decision only: INCLUDE, EXCLUDE, or UNCERTAIN. INCLUDE if the record appears potentially eligible. UNCERTAIN whenever eligibility cannot be determined from the title/abstract, especially when pDST, clinical-isolate status, or reconstructable accuracy data are not reported. EXCLUDE only when an exclusion criterion is explicit. Use a deliberately sensitive approach; do not exclude merely because the abstract does not report sensitivity, specificity, TP, FP, FN, TN, sample size, or the exact tool name.

Full-text screening instructions

Evaluate each criterion separately. Quote the exact supporting text and provide PDF page/table/supplement location. Assign one primary exclusion reason only, using the first applicable reason in the prespecified hierarchy: E1 → E2 → E3 → E4 → E5 → E6 → E7 → E8 → E9. If evidence is incomplete, return UNCERTAIN and state what must be checked or requested from authors.

Required output — title/abstract

Record ID: Citation: Decision: INCLUDE / EXCLUDE / UNCERTAIN Primary reason code: Reason in one sentence: Key evidence from title/abstract: Human verification required: YES

Required output — full text

Record ID: Citation: Population eligible: YES / NO / UNCERTAIN — evidence and page WGS index test eligible: YES / NO / UNCERTAIN — evidence and page Resistance-prediction method identifiable: YES / NO / UNCERTAIN — evidence and page Phenotypic reference standard present: YES / NO / UNCERTAIN — evidence and page Clinical isolates/specimens eligible: YES / NO / UNCERTAIN — evidence and page 2×2 data extractable/reconstructable: YES / NO / UNCERTAIN — evidence and

table/page Potential overlap with another report: YES / NO / UNCERTAIN Final recommendation: INCLUDE / EXCLUDE / UNCERTAIN Primary exclusion code, if excluded: Exact exclusion reason: Information requiring human verification:

Safety and integrity rules

- Never invent missing study details, counts, page numbers, quotations, tool names, or reference standards.
- Never infer pDST solely from words such as "validated," "benchmark," or "accuracy."
- Never treat genotypic consensus or another sequencing method as phenotypic reference standard unless the protocol explicitly permits it.
- Never make the final inclusion decision without human review.
- Preserve an audit trail containing prompt version, model/version, date, record ID, input text, output, human decision, reviewer name, and reason for any override.

12. AI Audit-Trail Fields

- Record ID
- Database and export batch
- Citation
- Screening stage
- Input text provided to AI
- Prompt version
- Model/provider and model version
- AI run date/time
- AI decision
- AI reason code
- AI evidence/quotation
- Human reviewer 1 decision
- Human reviewer 2 decision
- Consensus decision
- Override reason
- Reviewer name/date

13. Suggested Manuscript Wording (Once Search Strategies Are Executed or Disclosed as Reconstructed)

Recommended Methods text:

"MEDLINE via PubMed, Embase, Web of Science Core Collection, and the Cochrane Central Register of Controlled Trials (CENTRAL) were systematically searched for records published from 1 January 2000 through 28 January 2026. The original historical search strings and result counts were not preserved in a recoverable working file; database-specific strategies were therefore reconstructed from the registered review question and eligibility criteria and are provided for transparency, though they have not been re-executed against the live databases. The historical record counts reported in the PRISMA flow diagram are confirmed by the authors as

accurate contemporaneous results of the original screening process. No language or publication-type restrictions were applied at the search stage. AI-assisted screening was used only as a decision-support tool under a version-controlled prompt; all records were independently re-examined by two human reviewers, and all final decisions and exclusions were human-verified. The reconstructed search strategies and AI prompt are provided in Supplementary File S1."

14. Official Syntax Sources

https://pubmed.ncbi.nlm.nih.gov/help/

https://www.elsevier.support/embase/answer/how-do-i-search-in-embase

https://www.elsevier.support/embase/answer/what-field-codes-can-i-use-in-embase

https://webofscience.help.clarivate.com/en-us/Content/wos-core-collection/woscc-search-field-tags.htm

https://www.cochranelibrary.com/help/search
