# Supplementary material for: Algorithmic Determinants of Performance Heterogeneity in Whole-Genome Sequencing-Based Prediction of Drug Resistance in Mycobacterium tuberculosis: A Systematic Review and Meta-Analysis
Source: Microorganisms. 2026 Aug 18;14(8):1824. doi: 10.3390/microorganisms14081824 (PMC13515934; doi:10.3390/microorganisms14081824)
Supplement: Supplementary file 1 [file microorganisms-14-01824-s001.zip › Supplementary_File_S2.pdf]

PRISMA-DTA CHECKLIST

Algorithmic Determinants of Performance Heterogeneity in Whole-Genome Sequencing-Based Prediction of Drug Resistance in Mycobacterium tuberculosis: A Systematic Review and Meta-analysis

**Section/Topic**	**#**	**PRISMA-DTA Checklist Item**	**Reported in manuscript (section / line no.)**
TITLE / ABSTRACT			
Title	1	Identify the report as a systematic review (+/- meta-analysis) of diagnostic test accuracy (DTA) studies.	Title page (line 1-3)
Abstract	2	See PRISMA-DTA for Abstracts.	Abstract, lines 30-56
INTRODUCTION			
Rationale	3	Describe the rationale for the review in the context of what is already known.	Introduction, para. 4-5, lines 91-100
Clinical role of index test	D1	State the scientific and clinical background, including the intended use and clinical role of the index test, and the rationale for minimally acceptable test accuracy.	Introduction, para. 1-3, lines 57-90
Objectives	4	Provide an explicit statement of question(s) addressed in terms of participants, index test(s), and target condition(s).	Introduction, final para., lines 101-106; PROSPERO Review objectives
METHODS			
Protocol and registration	5	Indicate if a review protocol exists, where it can be accessed, and registration information.	Methods 2.1, lines 108-112; PROSPERO CRD420261342739 (registered 10 Apr 2026)
Eligibility criteria	6	Specify study, participant, index test, reference standard, target condition, and study-design characteristics used as eligibility criteria, with rationale.	Methods 2.3, lines 133-143; PROSPERO Eligibility Criteria section
Information sources	7	Describe all information sources and date last searched.	Methods 2.2, lines 120-128
Search	8	Present full search strategies for all databases and sources, including limits used.	Methods 2.2 (summary); full reconstructed strategies for all four databases, disclosed as reconstructed rather than original, are provided in Supplementary File S1
Study selection	9	State the process for selecting studies (screening, eligibility, included in review / meta-analysis).	Methods 2.3, lines 133-143; Figure 1 (PRISMA flow diagram)
Data collection process	10	Describe method of data extraction and processes for obtaining/confirming data from investigators.	Methods 2.4, lines 144-154
Definitions for data	11	Provide definitions used in data extraction and	Methods 2.1 (dataset definition), lines 117-119; Methods 2.4, lines 144-

extraction		classification of target condition(s), index test(s), reference standard(s), and other characteristics.	154
Risk of bias and applicability	12	Describe methods for assessing risk of bias and applicability concerns.	Methods 2.5, lines 155-161
Diagnostic accuracy measures	13	State the principal diagnostic accuracy measure(s) reported and the unit of assessment.	Methods 2.6, lines 163-168 (sensitivity/specificity, per-dataset)
Synthesis of results	14	Describe methods of handling data, combining study results, and describing variability, incl. handling of multiple thresholds/readers/reference standards.	Methods 2.6, lines 163-177
Meta-analysis	D2	Report the statistical methods used for meta-analyses, if performed.	Methods 2.6 (bivariate random-effects model jointly estimating sensitivity and specificity, fitted via metafor::rma.mv(), R 4.3.3; primary analytic unit is 7 independent tools; a univariate sensitivity analysis is also reported; the mada package was not used at any stage)
Additional analyses	16	Describe methods of additional analyses (sensitivity/subgroup/meta-regression), indicating which were pre-specified.	Methods 2.6, lines 169-177 (subgroup analyses, meta-regression, leave-one-out, Deeks' test — all pre-specified per PROSPERO)
RESULTS			
Study selection	17	Provide numbers of studies screened, assessed, and included, with reasons for exclusion at each stage, ideally with a flow diagram.	Results 3.1, lines 179-191; Figure 1
Study characteristics	18	For each included study, provide citations and key characteristics.	Results Section 3.1; Table 1; Supplementary Table S3 (detailed full-text characteristics are verified for the seven quantitative studies; stated data gaps remain for the other ~~20~~12 included studies).
Risk of bias and applicability	19	Present evaluation of risk of bias and applicability concerns for each study.	Updated QUADAS-2 workbook. QUADAS-2 was independently assessed by two reviewers for all ~~27~~19 included studies; disagreements were resolved by consensus. Domain-level and reviewer-level assessments, including consensus judgements, are provided in the workbook.
Results of individual studies	20	For each analysis, report 2x2 data (TP, FP, FN, TN) with accuracy estimates and CIs, ideally with forest/ROC plot.	Results Section 3.2; Table 2; Supplementary Table S1; Figure 2 (diagnostic-accuracy space and drug-specific pooled estimates).
Synthesis of results	21	Describe test accuracy including variability; if meta-analysis performed, include results and CIs.	Results 3.2-3.5, lines 201-236; Figures 2-3
Additional analysis	23	Give results of additional analyses	Results 3.4-3.6, lines 215-239; Figures 3-4

		(sensitivity/subgroup/meta-regression), if done.	
DISCUSSION			
Summary of evidence	24	Summarize the main findings, including strength of evidence.	Discussion, para. 1, lines 240-251
Limitations	25	Discuss limitations from included studies and from the review process.	Discussion, para. 12, lines 328-340
Conclusions	26	Provide a general interpretation of results in context of other evidence; discuss implications for research and practice.	Discussion, final para. and Conclusions, lines 341-351
FUNDING			
Funding	27	Describe sources of funding and other support and the role of funders.	Funding statement, lines 361-367

Adapted from: McInnes MDF, Moher D, Thombs BD, McGrath TA, Bossuyt PM, the PRISMA-DTA Group. Preferred Reporting Items for a Systematic Review and Meta-analysis of Diagnostic Test Accuracy Studies: The PRISMA-DTA Statement. JAMA. 2018;319(4):388-396. doi:10.1001/jama.2017.19163.

Note: Locations are identified by manuscript section. Exact line numbers may change during production; all cited sections refer to the final proof version.
