# Supplementary material for: Algorithmic Determinants of Performance Heterogeneity in Whole-Genome Sequencing-Based Prediction of Drug Resistance in Mycobacterium tuberculosis: A Systematic Review and Meta-Analysis
Source: Microorganisms. 2026 Aug 18;14(8):1824. doi: 10.3390/microorganisms14081824 (PMC13515934; doi:10.3390/microorganisms14081824)
Supplement: Supplementary file 1 [file microorganisms-14-01824-s001.zip › Supplementary_File_S3/Corrected_Statistical_Reanalysis_Report.docx]

**Corrected Statistical Reanalysis Report**

*Manuscript ID: microorganisms-4395033*

**1. Analytical dataset and purpose**

The quantitative dataset contained 28 drug-level 2 × 2 tables for rifampicin, isoniazid, ethambutol, and pyrazinamide, contributed by seven bioinformatic tools. The four drug-specific tables within each tool are correlated and were therefore not treated as 28 independent studies in the primary analysis.

For the primary overall analysis, the four first-line drug-specific 2 × 2 tables were summed within each tool to produce one composite table per tool. A bivariate random-effects model was then fitted to the seven tool-level composite tables. This report supersedes prior 28-record univariate overall estimates as the primary result.

**2. Primary tool-level bivariate analysis**

The primary model was a bivariate random-effects logistic regression fitted by REML, with tool as the analytic unit (n = 7). The model jointly estimated sensitivity and specificity and their between-tool correlation.

| Measure | Estimate | 95% CI | Model |
| --- | --- | --- | --- |
| Sensitivity | 0.930 | 0.907–0.948 | Tool-level bivariate REML |
| Specificity | 0.962 | 0.929–0.981 | Tool-level bivariate REML |
| Sensitivity–specificity correlation | 0.304 | Not applicable | Between-tool correlation |

The reported overall estimates in the manuscript, Figure 2, and Supplementary Table S2 are therefore sensitivity 0.930 (95% CI: 0.907–0.948) and specificity 0.962 (95% CI: 0.929–0.981).

**3. Secondary drug-specific analyses**

Drug-specific estimates were generated separately using univariate REML models across the seven tools for each drug. These analyses describe drug-level variation and are not the primary overall bivariate estimate.

| Drug | Sensitivity (95% CI) | Specificity (95% CI) |
| --- | --- | --- |
| Rifampicin | 0.960 (0.934–0.976) | 0.976 (0.958–0.986) |
| Isoniazid | 0.933 (0.906–0.953) | 0.982 (0.963–0.991) |
| Ethambutol | 0.913 (0.886–0.934) | 0.932 (0.878–0.963) |
| Pyrazinamide | 0.860 (0.800–0.904) | 0.965 (0.920–0.985) |

**4. Exploratory algorithm-category comparison**

At the tool level, the estimated pooled sensitivities were 0.920 for rule-based tools, 0.899 for machine-learning tools, and 0.951 for hybrid tools. The hybrid-versus-rule-based sensitivity contrast was 0.533 on the logit scale (95% CI: 0.049–1.018; p = 0.031); the machine-learning-versus-rule-based contrast was −0.256 (95% CI: −0.773 to 0.262; p = 0.333). These estimates are descriptive and hypothesis-generating only. Algorithm category is completely confounded with tool identity, training data, mutation catalogue version, and validation population.

**5. Provenance sensitivity analysis**

The source-data table classified 8 records as direct, 14 as reconstructed, and 6 as provisional/reconstructed. After exclusion of all six provisional/reconstructed records, 22 drug-level records from six tools remained. The corresponding tool-level bivariate estimates were sensitivity 0.937 (95% CI: 0.914–0.955) and specificity 0.973 (95% CI: 0.949–0.986).

**6. Exploratory funnel-plot assessment**

The Deeks’ funnel plot asymmetry test was run on the 28 drug-level records only as an exploratory assessment. The slope coefficient was −20.34 (p = 0.765). Because the four records within each tool are correlated and there are only seven independent tools, this non-significant result should not be interpreted as evidence of an absence of publication bias.

**7. Reproducibility**

All analyses were run in R version 4.5.2 with metafor version 4.8-0. The final scripts, extracted 2 × 2 data, and Figure 2 source-data outputs are provided in Supplementary File S3.
